# Spontaneous hemothorax in Neurofibromatosis type 1 with a giant intrathoracic meningocele and intracystic hematoma: Diagnostic value of postmortem CT

**DOI:** 10.1016/j.radcr.2026.03.039

**Published:** 2026-05-06

**Authors:** Yohei Ikebe, Taisuke Harada, Zen-ichi Tanei, Nanase Okazaki, Toshiya Osanai, Shinya Tanaka, Kohsuke Kudo

**Affiliations:** a Department of Diagnostic and Interventional Radiology, Hokkaido University Hospital, Sapporo, Hokkaido, Japan; b Center for Cause of Death Investigation, Faculty of Medicine and Graduate School of Medicine, Hokkaido University, Sapporo, Hokkaido, Japan; c Department of Diagnostic Imaging, Faculty of Medicine and Graduate School of Medicine, Hokkaido University, Sapporo, Hokkaido, Japan; d Department of Cancer Pathology, Faculty of Medicine, Hokkaido University, Sapporo, Hokkaido, Japan; e Department of Surgical Pathology, Hokkaido University Hospital, Sapporo, Hokkaido, Japan; f Department of Neurosurgery, Faculty of Medicine and Graduate School of Medicine, Hokkaido University, Sapporo, Hokkaido, Japan; g Institute for Chemical Reaction Design and Discovery (WPI-ICReDD), Hokkaido University, Sapporo, Hokkaido, Japan

**Keywords:** Neurofibromatosis type 1, Intrathoracic meningocele, Intra-meningocele hemorrhage, Hemothorax, Postmortem CT

## Abstract

We report a case of spontaneous hemothorax in a 48-year-old woman with Neurofibromatosis type 1 (NF1), presumed to result from rupture of an intrathoracic meningocele. She had been hospitalized for treatment of an intracranial carotid artery aneurysm and underwent endovascular embolization. Preoperative body CT demonstrated a meningocele protruding into the left thoracic cavity at the level of the T3/T4 vertebrae. On postoperative day 6, she experienced sudden cardiopulmonary arrest and died. Postmortem CT revealed a massive left hemothorax and an acute high-density hematoma within the intrathoracic meningocele. Autopsy findings were consistent with hemothorax likely caused by rupture of the meningocele. NF1 is associated with intrathoracic meningoceles and vascular fragility. The acute hematoma seen within the meningocele on postmortem CT suggested meningocele rupture as the source of bleeding, illustrating a rare but characteristic complication of NF1.

## Introduction

Neurofibromatosis type 1 (NF1) is an autosomal dominant genetic disorder caused by mutations in the NF1 tumor suppressor gene, with an estimated prevalence of 1 in 2,000 to 3,000 individuals [1].

In addition to neoplastic manifestations—most notably neurofibromas—NF1 also leads to non-neoplastic manifestations, such as skeletal dysplasia, mesenchymal dysplasia and vasculopathy [2,3].

Skeletal and mesenchymal dysplasia are major causes of spinal meningoceles, which is the protrusion of the dura mater and cerebrospinal fluid (CSF) through a defect in the bone. Furthermore, vasculopathy-related vascular fragility may result in spontaneous bleeding in various organs.

There have been several reports of spontaneous hemothorax in NF1 patients [[4], [5], [6], [7]], but few have described an association with intrathoracic meningocele.

In this report, we present a fatal case of hemothorax suspected to be caused by rupture of an intrathoracic meningocele, which is a rare condition, with detailed findings of postmortem CT and autopsy.

## Case report

The patient was a 48-year-old woman undergoing treatment for Neurofibromatosis type 1 (NF-1). Her past medical history included partial resection of a neurofibroma in the right neck extending to the parapharyngeal space with tracheostomy, and cranioplasty for a bony defect in the right posterior cranial fossa. There was no significant family history, including NF-1.

**Fig. 1:**
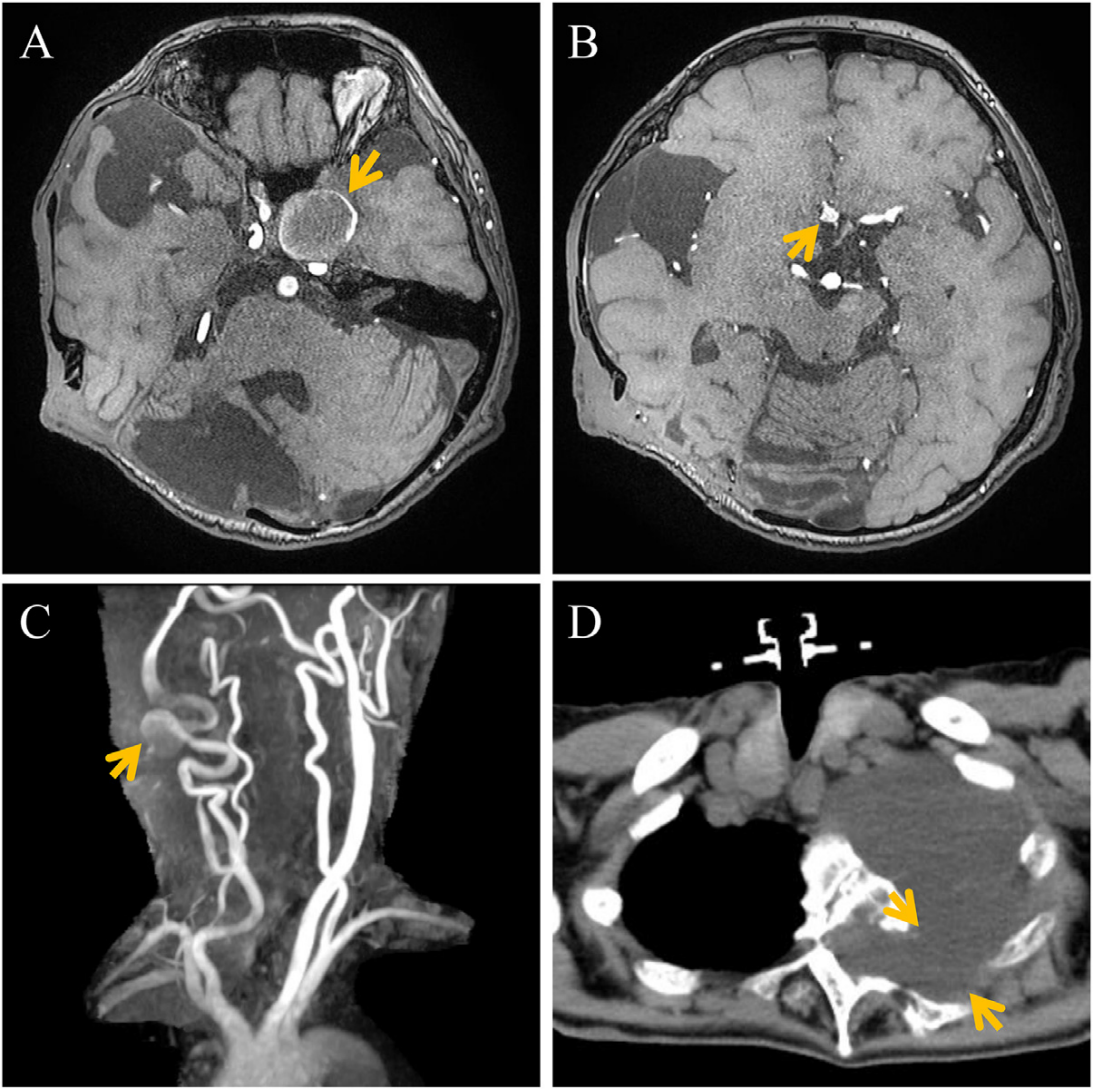
Preoperative images. (A-C) MR angiography, (D) Chest CT. (A) Left internal carotid artery (ICA) giant aneurysm, strongly compressing the left optic nerve and the cavernous sinus (arrow), a bony defect of the clivus and right posterior cranial fossa, an old infarction in the right cerebellum was observed. (B, C) Anterior communicating artery aneurysm (B, arrow) and cervical segment of the right ICA aneurysm (C, arrow) and marked arterial tortuosity were seen. (D) A meningocele protruding into the left thoracic cavity at the T3-4 level was identified (arrow). The left T3-4 intervertebral foramen was expanded secondary to a meningocele.

She initially noticed visual disturbances in the left eye. After about 1 year, she developed left eye pain, ptosis, and diplopia. Brain MRI revealed a giant aneurysm, approximately 25 mm in diameter, located at the left internal carotid artery (ICA) siphon, strongly compressing the left optic nerve and the cavernous sinus (Fig. 1A).

Additional findings on brain MRI and CT included a 5 mm aneurysm at the anterior communicating artery (Fig. 1B), a 10 mm aneurysm at the cervical segment of the right extracranial ICA with marked arterial tortuosity (Fig. 1C), an old infarction in the right cerebellum, and a bony defect of the clivus with a corresponding meningocele (Fig. 1A). On body CT, a meningocele protruding into the left thoracic cavity at the T3-4 level was identified (Fig. 1D). The left T3-4 intervertebral foramen was expanded secondary to a meningocele. The intrathoracic meningocele measured 128 mm at its maximal diameter, whereas it had measured 60 mm on CT 15 years earlier, demonstrating gradual enlargement. No obvious symptoms were attributable to the meningocele.

Compression of cranial nerves by the left ICA giant aneurysm was considered the cause of her symptoms. Given its large size and presumed high risk of rupture, she was admitted for aneurysm treatment. A superficial temporal artery to middle cerebral artery bypass was first performed, followed by endovascular coil occlusion of the left ICA aneurysm on the next day.

After the coiling procedure, multiple small cerebral infarctions developed in both hemispheres. These were managed conservatively, including antithrombotic therapy, and her condition remained otherwise stable.

On postoperative day 6, early in the morning, she was found unresponsive with dilated pupils and a Glasgow Coma Scale score of 3 (E1V1M1). Prodromal symptoms could not be confirmed, and no subtle clinical changes were observed prior to the event. She experienced cardiopulmonary arrest shortly thereafter. Despite approximately 30 minutes of resuscitation efforts, she was pronounced dead.

**Fig. 2:**
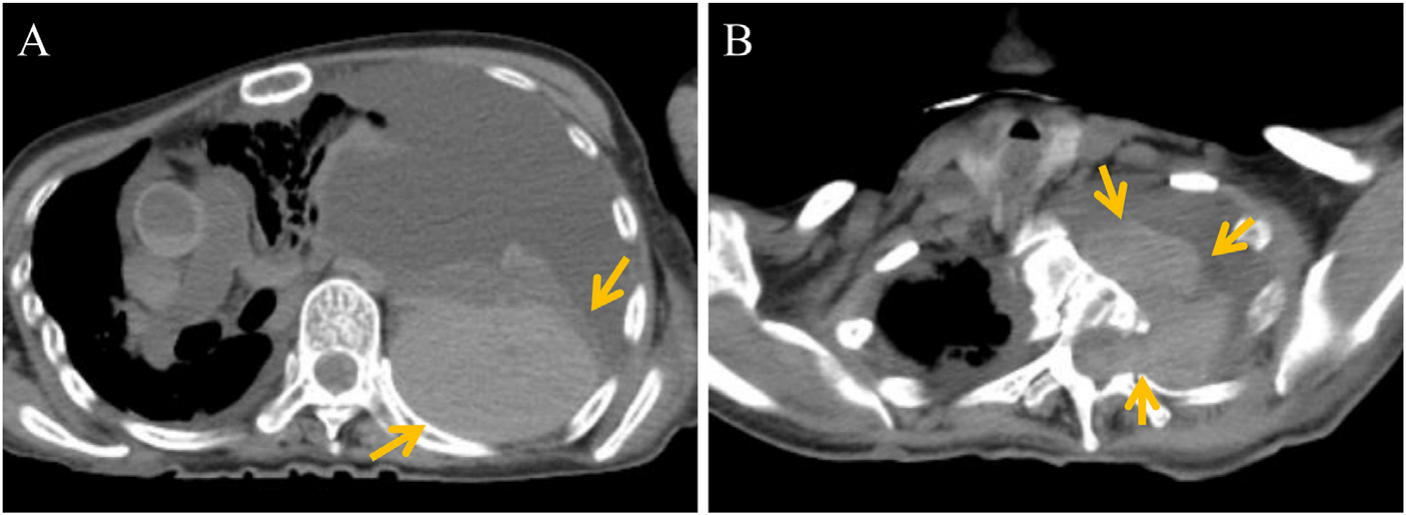
Postmortem CT. Postmortem CT revealed intrathoracic fluid accumulation and high-attenuation hematoma predominantly located in the dorsal aspect of the left thoracic cavity (A, arrow). The meningocele in the left thoracic cavity was also filled with high-attenuation hematoma (B, arrow), suggesting the presence of antemortem hemorrhage. Mediastinal shift to the right was observed due to the left-sided hemothorax.

With family consent, postmortem CT and autopsy were conducted to investigate the cause of death. Postmortem CT before autopsy was obtained approximately 1 hour after death. It revealed fluid accumulation with high attenuation predominantly in the dorsal aspect of the left thoracic cavity (Fig. 2A), suggesting a large left hemothorax. A mass-like hematoma was present, indicating bleeding occurred before death, since coagulation is a vital reaction. The meningocele in the left thoracic cavity at the T3-4 level was also filled with high-attenuation structure (Fig. 2B), suspected to be hematoma.

**Fig. 3:**
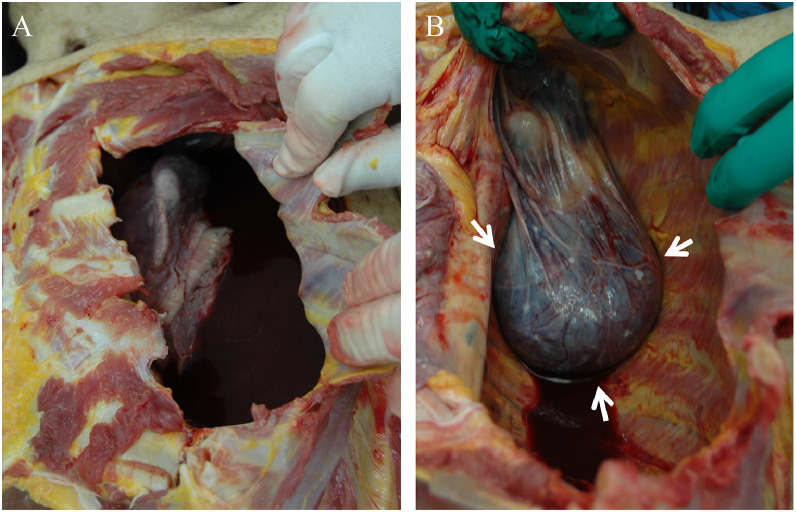
Autopsy findings. Autopsy confirmed about 1,900 mL of blood in the left thoracic cavity and a ruptured meningocele containing an acute hematoma (B, arrow).

The autopsy was performed approximately 5 hours after death. Autopsy confirmed approximately 1,900 mL of blood in the left thoracic cavity (Fig. 3A). The meningocele protruding into the thoracic cavity was observed, accompanied by deformation of the adjacent thoracic vertebrae. An acute hematoma was contained within the meningocele, consistent with the postmortem CT findings, and the rupture of the meningocele into the thoracic cavity was confirmed (Fig. 3B). The exact location of the vascular rupture responsible for the bleeding could not be identified. Small amounts of hematoma were seen in the spinal subdural space at the cervical level and within the fourth ventricle, suggesting spread of blood through the cerebrospinal fluid space. There were no findings suggesting rupture of the previously treated left ICA aneurysm and other aneurysms or other alternative causes of death. The cause of death was concluded to be a left hemothorax, likely due to rupture of the intrathoracic meningocele.

## Discussion

Intrathoracic meningocele is a CSF-filled sac resulting from the protrusion of the meninges into the thoracic cavity. This occurs through either an enlarged intervertebral foramen or a bony defect in the thoracic spine. The condition is rare and usually found in patients with genetic syndromes such as NF1 and Marfan syndrome. According to Andrade et al., [8] 69% of thoracic meningoceles were associated with NF1, while only 22% appeared without any underlying disorder. Symptoms are often absent; however, some patients may experience symptoms depending on the size and location of the meningocele. On CT, they appear as well-defined paravertebral masses with low attenuation, corresponding to cerebrospinal fluid signal intensity on MRI. CT myelography shows intrathecal contrast material filling these spaces [3]. Rupture of an intrathoracic meningocele is a possible complication and can result in a large pleural effusion [9]. Ryttman [10] reported a patient diagnosed with meningocele rupture by performing myelography. In the present case, anatomical examination confirmed disruption of the meningocele, which likely led to the hemothorax. Although its size increased over time, the patient remained asymptomatic until rupture, so conservative management was preferred to surgery.

NF1 is known to cause fragile blood vessels and related vascular complications, collectively referred to as NF1 vasculopathy [11]. This arterial fragility is thought to result from dense proliferation of smooth muscle cells within the arterial walls, accompanied by medial cystic degeneration, fragmentation of the elastic layer, and ischemia caused by compression of the vessels’ nutrient supply due to tumor infiltration [12]. These changes are not present in all arteries, and differentiating between normal and affected vessels is difficult [13]. Fragile arteries may bleed from unusual sites, making it difficult to identify the origin of hemorrhage, and management can also become complex. Arterial stenosis is the most frequent vascular finding, but aneurysms, dissections, ruptures, and arteriovenous malformations have also been observed [3,11]. Although the exact prevalence of the vasculopathy remains unknown, vasculopathy is the second leading cause of death in NF-1, surpassed only by malignancies [14]. In this case, intracranial and cervical aneurysms, and marked arterial tortuosity were present, strongly suggesting NF1 vasculopathy. Although a ruptured vessel was not clearly identified, the vascular pathology likely contributed to the hemothorax.

Several previous cases have described spontaneous hemothorax in NF1 patients [[4], [5], [6], [7]]. These were often caused by rupture of intercostal arteries and subclavian arteries [7]. In addition to arterial fragility, mechanical stress from spinal deformities such as scoliosis may contribute to vascular rupture [15]. A few reports have documented hemothorax in the presence of intrathoracic meningoceles [5,6]. However, based on our literature review, no prior case was found in which a hematoma had formed within the meningocele itself. To our knowledge, this is the first report of hemothorax suspected to be due to rupture of an intrathoracic meningocele, although it is possible that chest compression caused the rupture of the meningocele, followed by the shift of the hematoma from the pleural cavity into the meningocele. Nonetheless, this possibility is considered limited, since the coagulated hematoma within the meningocele suggested antemortem hemorrhage. The coagulated hematoma was also identifiable as a high-attenuation structure without a fluid-fluid level on postmortem CT, allowing the pathophysiology to be inferred without autopsy. Additionally, the rupture site was smaller than the hematoma within the meningocele, and this also suggests that direct bleeding into the pleural space is more likely. Antithrombotic therapy administered after coil embolization of the aneurysm may have contributed to the development of the hemothorax.

We present a rare case of spontaneous hemothorax associated with rupture of an intrathoracic meningocele in an NF1 patient. When hemothorax and hematoma within a meningocele are observed, spontaneous hemothorax due to rupture of an intrathoracic meningocele should be considered.

## Patient consent

Written informed consent was obtained from the patient’s next of kin using a comprehensive consent form. All images have been anonymized for publication/use.

## Author contributions

Yohei Ikebe: Drafted the manuscript and performed autopsy-pathological correlation. Taisuke Harada: corresponding author and revised the manuscript. Kohsuke Kudo: Interpreted the imaging findings. Zen-ichi Tanei, Nanase Okazaki, and Shinya Tanaka: Performed autopsy. Toshiya Osanai: Provided clinical management and patient care.
